# Moral and Social Values in Evidence-Informed Deliberative Processes for Health Benefit Package Design: Comment on "Evidence-Informed Deliberative Processes for Health Benefit Package Design – Part II: A Practical Guide"

**DOI:** 10.34172/ijhpm.2022.7480

**Published:** 2022-12-28

**Authors:** Michael J. DiStefano

**Affiliations:** ^1^Department of Health Policy & Management, Bloomberg School of Public Health, Johns Hopkins University, Baltimore, MD, USA.; ^2^Berman Institute of Bioethics, Johns Hopkins University, Baltimore, MD, USA.

**Keywords:** Deliberation, Health Technology Assessment, Legitimacy, Ethics, Value Judgments, Accountability for Reasonableness

## Abstract

An evidence-informed deliberative process (EDP) is defined as "a practical and stepwise approach for health technology assessment (HTA) bodies to enhance legitimate health benefit package design based on deliberation between stakeholders to identify, reflect and learn about the meaning and importance of values, informed by evidence on these values." In this commentary, I discuss some considerations for EDPs that arise from acknowledging the difference between social and moral values. First, the best practices for implementing EDPs may differ depending on whether the approach is grounded in moral versus social values. Second, the goals of deliberation may differ when focused on moral versus social values. I conclude by offering some considerations for future research to support the use of EDPs in practice, including the need to assess how different approaches to appraisal (eg, more quantitative versus qualitative) impact perceptions of the value of deliberation itself.

 An evidence-informed deliberative process (EDP) is defined as “a practical and stepwise approach for health technology assessment (HTA) bodies to enhance legitimate health benefit package design based on deliberation between stakeholders to identify, reflect and learn about the meaning and importance of values, informed by evidence on these values.”^1^ The EDP framework provides helpful practical guidance for HTA bodies concerned with the legitimacy of their decision-making. The authors are right to note that the accountability for reasonableness (A4R) framework, which grounds the EDP framework, does not provide such practical guidance beyond identifying the key elements of a legitimate priority-setting process. In response, the authors have developed a stepwise approach for implementing EDPs grounded in A4R, illustrated with examples from national HTA bodies and the HTA literature. In this commentary, I discuss some considerations for EDPs that arise from the difference between social and moral values as well as some considerations for future research to support the use of EDPs in practice.

## Social Versus Moral Values

 When discussing values, the authors are particularly focused on the values underlying or comprising HTA decision criteria. Such values may reflect both the goals of HTA (eg, reduce suffering, reduce health inequities) and its methods (eg, endpoint selection when assessing effectiveness, different methods of cost-effectiveness analysis).^2^ In the definition of an EDP and throughout the elaboration of the framework, however, it is not clear whether the authors have in mind (1) values that are ultimately grounded in morality or (2) values that express purely descriptive preferences of the members or different elements of society. While the authors explicitly reference ‘social values’ several times, this term is not consistently understood or applied in the HTA and health priority-setting literature. For example, an influential conceptual framework for health priority setting defines social value judgments as “judgments made on the basis of the moral or ethical values of a particular society,” which is to say they “give particular form to ‘universal’ moral values.”^3^ Such values are ultimately justifiable through argument and according to moral theory and abstract moral concepts like justice. Alternative definitions refer to social values as “values that are considered important by the society that is affected by the decision-making process”^4^ or “broadly shared values in society which bear on the appropriate use and impact of the [health] technology.”^5^ Such values, defined in these ways, need not be justifiable according to moral theory; they may simply express the preferences or opinions of members of society. The key difference between these concepts is thus the source or principle of justification. While the former is justified through argument drawing on moral theory and concepts, the latter is justifiable through democratic principles. For purposes of clarity, I will refer to the former concept as ‘moral values’ (more specifically, the particular forms of ‘universal moral values’) and the latter as ‘social values.’ In addition to the importance of ensuring that all participants in EDPs are on the same page regarding the nature of specific values about which they are deliberating, there are several reasons why further theoretical development of EDPs and their implementation in practice should give careful attention to the difference between moral and social values.

 First, the best practices for implementing EDPs may differ depending on whether the approach is grounded in moral versus social values. For example, the process for defining decision criteria that draw on moral values will require engaging with key moral frameworks and moral concepts and arguments, not simply a “review of policy documents on national health strategies”^1^ as the authors describe in their discussion of this step. As an example of what such a process can look like, the South African Values and Ethics for Universal Health Coverage (SAVE-UHC) project recently developed a context-specific ethics framework for HTA analysis and appraisal in support of National Health Insurance in South Africa that is the product of multi-stakeholder deliberations grounded in reviews of both policy documents and relevant moral frameworks and concepts.^6^

 Second, the goals of deliberation may differ when focused on moral versus social values. The authors write the following about the goals of EDPs:

 “…stakeholders may *deepen their understanding* of their own preferences and those of others affected by decisions. They may *replace uninformed opinions* by views that are more rational and better supported by arguments and evidence, improving the quality of the decisions. There is good evidence that participants *learn* from deliberative engagement, including considering information that is contrary to their opinions and can change their opinions in line with this new information”^7^ [emphases added].

 To begin, improved understanding may be necessary, but not sufficient, as a goal for deliberation around moral values. Ultimately, for a value to be justifiable as a moral value it must be justifiable according to moral theory and concepts. Understanding one’s own or another’s values can therefore only be an initial step in moral deliberation, which additionally requires engaging with moral theory, concepts, and reasoning to establish moral justifiability. Often, social and moral values will overlap (ie, social values can be morally justifiable), but they can also diverge such that some social values will express unethical preferences (eg, racist or misogynistic views). A process explicitly committed to deliberating about moral values cannot simply accept social values that lack moral justification, even if they are widely held. Additionally, the goal of replacing uninformed opinions is described here primarily in terms of identifying instances where preferences rest on flawed evidence or bad reasoning about evidence. But identifying and correcting unjustified moral values will require more than examining the empirical evidence; as just described above, it will require engagement with moral theory, concepts, and argument. To provide a real-world example, the National Institute for Health and Care Excellence (NICE) states that it does not modify its approach to assessing the value of health technologies if a health condition has been caused by a person’s behavior.^8^ However, a number of studies have found that members of the public typically express a preference for allocating resources for health conditions that are not the result of lifestyle factors like smoking or alcohol consumption.^9^ In deliberations involving the public, NICE should therefore defend its reasons for rejecting this possible social value. In providing this defense, NICE may draw on different moral arguments — the importance of considering the social context that influences individual behaviors,^10^ the unjust focus on only certain behaviors or the potential for increasing inequity^11^ — to justify its position. Through such deliberation, participants may be pushed beyond mere understanding of an alternative view to question and explain whether their own view is morally justifiable, resulting in a deeper form of learning.

 In general, there is not much known about the potential unique impacts of moral deliberation on the quality of HTA decisions and on those who participate in the decision-making process. While there have been studies investigating the impact of moral reasoning exercises on individual preferences for healthcare priority-setting,^12,13^ these studies do not incorporate moral reasoning as part of structured deliberation with other individuals. Similarly, recent studies investigating the impact of structured deliberation on individuals involved in health priority-setting^14–18^ have not incorporated explicit discussion of moral values. The implementation of EDPs that are explicitly committed to identifying, reflecting, and learning about moral values (as opposed to social values) represents an opportunity for further study of this matter. For example, one potential outcome of moral deliberation in HTA could be a greater appreciation among participants for the pervasively moral nature of HTA processes and decision-making. That is, participants may come to understand that moral values are not only expressed in HTA by a small number of explicit “ethics” decision criteria, but (to give just one example) that they also undergird traditional decision criteria like cost-effectiveness in a variety of ways. The SAVE-UHC project described above is one example of such an approach. After developing the provisional ethics framework, researchers convened simulated appraisal committees to test the application of the ethics framework in structured deliberations aimed at reaching coverage recommendations for several hypothetical health technology cases.^6^

## Future Research to Support EDPs

 The authors call for future research to support the use of EDPs. I conclude by offering several considerations to help guide this future research. First, this latest development of the EDP framework emphasizes stakeholder participation as the ideal form of stakeholder involvement. The authors distinguish stakeholder participation from consultation and communication and argue that EDPs should be organized around participation and with efforts made to remove any barriers to effective participation^7^ (ie, barriers that could arise from power disparities or differences in expertise that exist between participants). Some of these barriers may relate to the appraisal committee size and composition, committee dynamics and the role of the chair, or the format of the appraisal meeting and decision-making, all features of HTA that are currently underexamined empirically.^19^ Designing studies with the objective of better understanding the relationship between these specific features of HTA and effective stakeholder participation should be one focus of the monitoring and evaluation activities proposed by the authors as EDPs are implemented around the world.

 Additionally, the authors acknowledge that “the use of EDPs is claimed to improve the legitimacy of benefit package design but so far only anecdotal evidence is available.”^1^ Indeed, Norman Daniels has made a similar point about the A4R framework, noting that most research has assessed whether its conditions are met in practice, but not whether meeting the conditions has the desired effects on legitimacy.^20^ Tackling this empirical question will require identifying appropriate outcome measures to indicate legitimacy. It may be prudent to look to other academic disciples for guidance. For example, researchers in political science have measured perceived legitimacy in terms of procedure acceptance, decision acceptance, and trust in the decision-makers.^21^

 Finally, an important insight the authors have gleaned from studying the implementation of EDPs over the past several years is that, “committee members seem to have a strong intuitive preference for the use of quantitative approaches to trade-off decision criteria.”^1^ This raises the possibility that the use of more qualitative appraisal processes might in fact undermine acceptance—and thus the perceived legitimacy—of EDPs from the perspective of such committee members and other stakeholders who share this intuitive preference. However, a possible outcome of engaging in different types of appraisal processes may be that participants learn about the relative advantages and disadvantages of these different approaches and update their initial preferences about how tradeoffs between decision criteria should best be assessed and managed. With this in mind, efforts to empirically assess the relationship between EDPs and perceived legitimacy should be longitudinal in nature and designed to understand how participant attitudes about the EDPs may evolve over time. Additionally, certain appraisal processes may be more or less suited to facilitating deliberation around moral versus social values. This should also be an important focus for future research on EDPs.

## Ethical issues

 Not applicable.

## Competing interests

 Author declares that he has no competing interests.

## Author’s contribution

 MJD is the single author of the paper.
